# (Tris{2-[(5-chloro-2-oxido­benzyl­idene-κ*O*)amino-κ*N*]eth­yl}amine-κ*N*)­ytterbium(III): crystal structure and Hirshfeld surface analysis

**DOI:** 10.1107/S2056989016013748

**Published:** 2016-09-05

**Authors:** See Mun Lee, Kong Mun Lo, Sang Loon Tan, Edward R. T. Tiekink

**Affiliations:** a Research Centre for Crystalline Materials, Faculty of Science and Technology, Sunway University, 47500 Bandar Sunway, Selangor Darul Ehsan, Malaysia

**Keywords:** crystal structure, lanthanide, coordination complex, hepta­dentate ligand, Hirshfeld surface analysis

## Abstract

The Yb^III^ atom in the title complex, [Yb(C_27_H_24_Cl_3_N_4_O_3_)] [systematic name: (2,2′,2′′-{(nitrilo)­tris­[ethane-2,1-di­yl(nitrilo)­methylyl­idene]}tris­(4-chloro­phenolato)ytterbium(III)], is coordinated by a trinegative, hepta­dentate ligand and exists within an N_4_O_3_ donor set, which defines a capped octa­hedral geometry whereby the amine N atom caps the triangular face defined by the three imine N atoms. The packing features supra­molecular layers that stack along the *a* axis, sustained by a combination of aryl-C—H⋯O, imine-C—H⋯O, methyl­ene-C—H⋯π(ar­yl) and end-on C—Cl⋯π(ar­yl) inter­actions. A Hirshfeld surface analysis points to the major contributions of C⋯H/ H⋯C and Cl⋯H/H⋯Cl inter­actions (along with H⋯H) to the overall surface but the Cl⋯H contacts are at distances greater than the sum of their van der Waals radii.

## Abstract 2

The title compound features an amine-N-capped octa­hedral coordination geometry for Yb^III^ defined by an N_4_O_3_ donor set. The packing features supra­molecular layers sustained by C—H⋯O, C—H⋯π(ar­yl) and C—Cl⋯π(ar­yl) inter­actions.

## Chemical context   

Despite being less studied than transition metal complexes, the crystal chemistry of lanthanides is rich and diverse as their complexes can display various coordination numbers and geometries that are not readily predicted (Salehzadeh *et al.*, 2010 ▸). In recent years, inter­est in the coordination chemistry of lanthanides has increased owing, for example, to the mol­ecular dynamics exhibited by their complexes (Pedersen *et al.*, 2014 ▸). Further, lanthanide-based luminescent compounds are potentially useful materials for the fabrication of organic light-emitting devices (OLEDs) due to their ability to exhibit sharp emission bands, high colour purity and long-lived emission states (Ahmed *et al.*, 2016 ▸; Bünzli *et al.*, 2015 ▸). In the context of the present report, tripodal and tri-anionic hepta­dentate ligands, having tertiary-amine, three neutral imine and three phenolate donors, leading to a potential N_4_O_3_ donor set, are of inter­est in coordination chemistry due to the cavity size they define and owing to the relative rigidity of the ligand. Being large and having seven potential donor atoms, these ligands are capable of coordinating lanthanides even if the atomic sizes of lanthanides are greater in comparison to their transition metal counterparts (Yang *et al.*, 1995 ▸). Indeed, from the literature, several tripodal lanthanide complexes have been successfully characterized and described (Liu *et al.*, 1992 ▸; Bernhardt *et al.*, 2001 ▸; Kanesato *et al.*, 2001**a* ▸,b* ▸, 2004 ▸; Hu *et al.*, 2015 ▸). As part of an on-going study, the crystal and mol­ecular structures of the title hepta­dentate Yb^III^ complex (I) is described herein along with an analysis of its Hirshfeld surface.
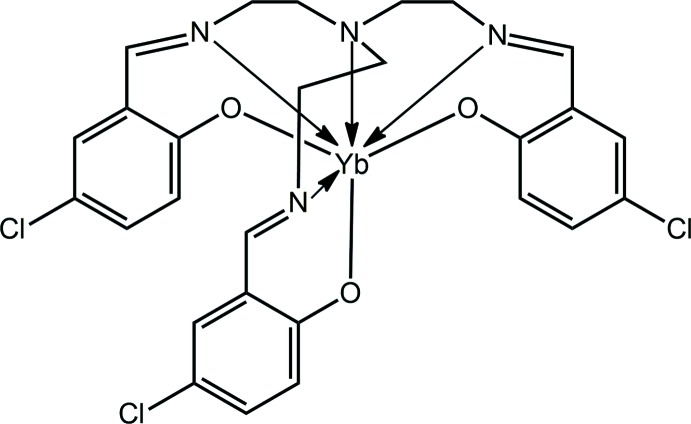


## Structural commentary   

**Figure 1:**
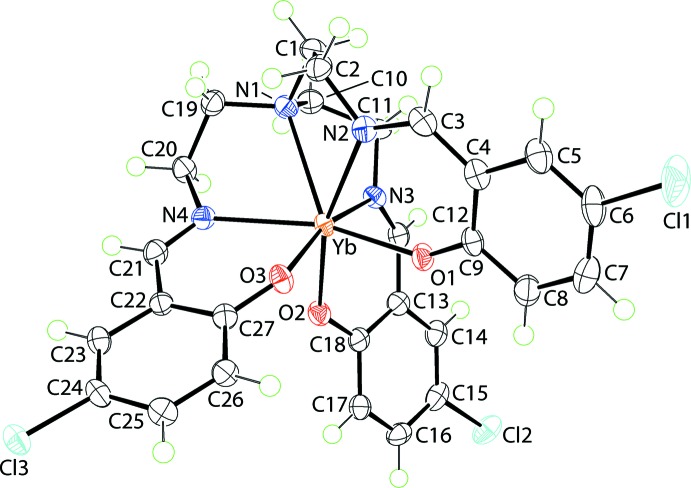
Mol­ecular structure of (I), showing the atom-labelling scheme and displacement ellipsoids at the 70% probability level.

The mol­ecular structure of (I) is shown in Fig. 1 ▸ and selected geometric parameters are collected in Table 1 ▸. The tris{[5-chloro­salicyl­idene)amino]­eth­yl}amine) tri-anion coordinates in a hepta­dentate mode, utilizing the three phenolate-oxygen, three imine-nitro­gen and tertiary amine-nitro­gen atoms. The coordination geometry is based on a amine-N-capped octa­hedron with the amine-nitro­gen atom capping the triangular face defined by the three imine-nitro­gen atoms. Supporting this assignment are the observations that the Yb—O bond lengths are significantly shorter than the Yb—N(imine) bonds which, in turn, are shorter than the Yb—N(amine) bond length, Table 1 ▸. The dihedral angle between the phenolate-O_3_ and imine-N_3_ faces is 3.86 (6)°, indicating a parallel disposition. There is a range in the Yb—O bond lengths, *i.e*. >0.02 Å, with the shortest Yb—O1 bond being *trans* to the most loosely bound imine-N4 atom, and the longest Yb—O2 bond being *trans* to most tightly held imine-N2 atom, Table 1 ▸. The six-membered chelate rings adopt different conformations. Thus, the O1-chelate ring is essentially planar (r.m.s. deviation of the six fitted atoms = 0.0377 Å), with the maximum deviation of 0.0672 (14) Å being for atom O1. The O2-chelate ring is considerably less planar (r.m.s. deviation = 0.0551 Å) for the non-Yb atoms with the maximum deviation being 0.0850 (16) Å for the C12 atom, with the Yb atom, the flap atom in an envelope conformation, lying 0.762 (3) Å out of the least-squares plane defined by the remaining atoms. An inter­mediate geometry is found for the O3-chelate ring, also an envelope, with the Yb flap atom lying 0.524 (3) Å out of the plane of the remaining atoms (r.m.s. deviation = 0.0376 Å). When viewed down the amine-N–Yb vector, the ethyl­ene bridges are in the same orientation as are the benzene rings, and the organic residues splay outwards to define the shape of a capped cone.

## Supra­molecular features   

**Figure 2:**
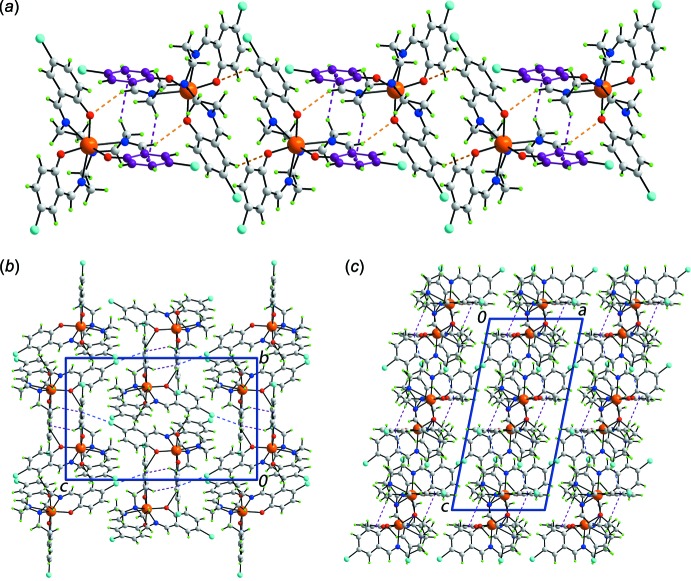
The packing in (I), showing (*a*) a helical supra­molecular chain along the *b* axis, (*b*) a supra­molecular layer in the *bc* plane and (*c*) a view in perspective down the *b* axis, The aryl-C—H⋯O, imine-C—H⋯O (orange), methyl­ene-C—H⋯π(ar­yl) (purple) and C—Cl⋯π(ar­yl) inter­actions (blue) are shown as dashed lines.

In the absence of conventional hydrogen bonding, the packing in (I) is sustained by a range of weak inter­actions, Table 2 ▸. Aryl-C—H⋯O and imine-C—H⋯O inter­actions assemble mol­ecules into supra­molecular helical chains propagating along the *b* axis, Fig. 2 ▸ *a*. These are reinforced by methyl­ene-C—H⋯π(ar­yl) contacts, also shown in Fig. 2 ▸ *a*. Chains are connected into supra­molecular layers in the *bc* plane by end-on C—Cl⋯π(ar­yl) inter­actions, Fig. 2 ▸ *b*. Layers stack along the *a* axis with no directional inter­actions between them, Fig. 2 ▸ *c* (Spek, 2009 ▸).

## Analysis of the Hirshfeld surfaces   

The close contacts present in the crystal of (I) were studied by mapping the Hirshfeld surface over the *d*_norm_ contact distances within the range of −0.18 to 1.65 Å through calculation of the inter­nal (*d*_i_) and external (*d*_e_) distances of a particular Hirshfeld surface point to its nearest nucleus (McKinnon *et al.*, 2007 ▸; Spackman & Jayatilaka, 2009 ▸), while the two-dimensional fingerprint plots of the corresponding close contacts were produced through the plot of *d*_e_ *vs d*_i_ (Spackman & McKinnon, 2002 ▸). All analyses were performed using *Crystal Explorer* (Wolff *et al.*, 2012 ▸). The distances involving hydrogen atoms were normalized to the standard neutron-diffraction bond lengths.

**Figure 3:**
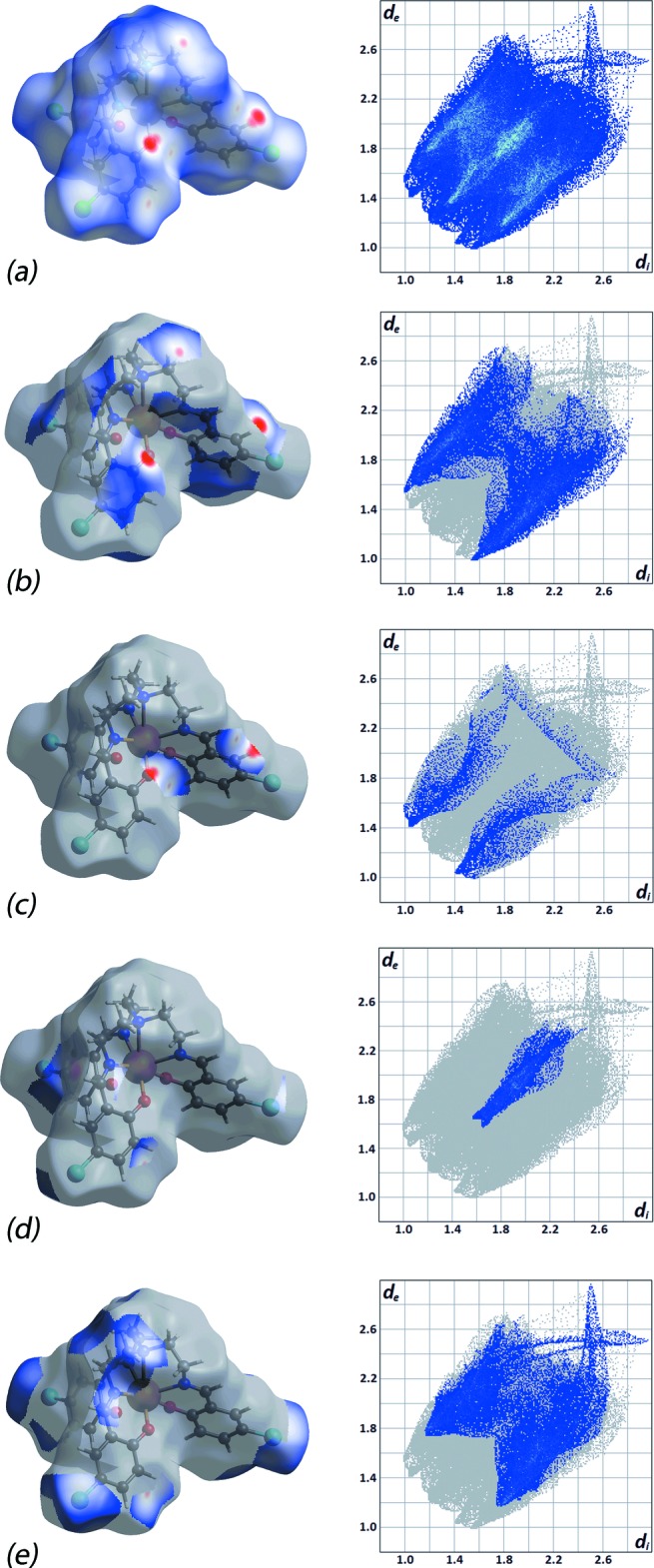
Comparison of (*a*) the complete Hirshfeld surface and full fingerprint plots for (I) and the corresponding *d*_norm_ surfaces and two-dimensional plots associated with (*b*) C⋯H/H⋯C, (*c*) O⋯H/H⋯O, (*d*) Cl⋯C/C⋯Cl and (*e*) Cl⋯H/H⋯Cl contacts.

**Figure 4:**
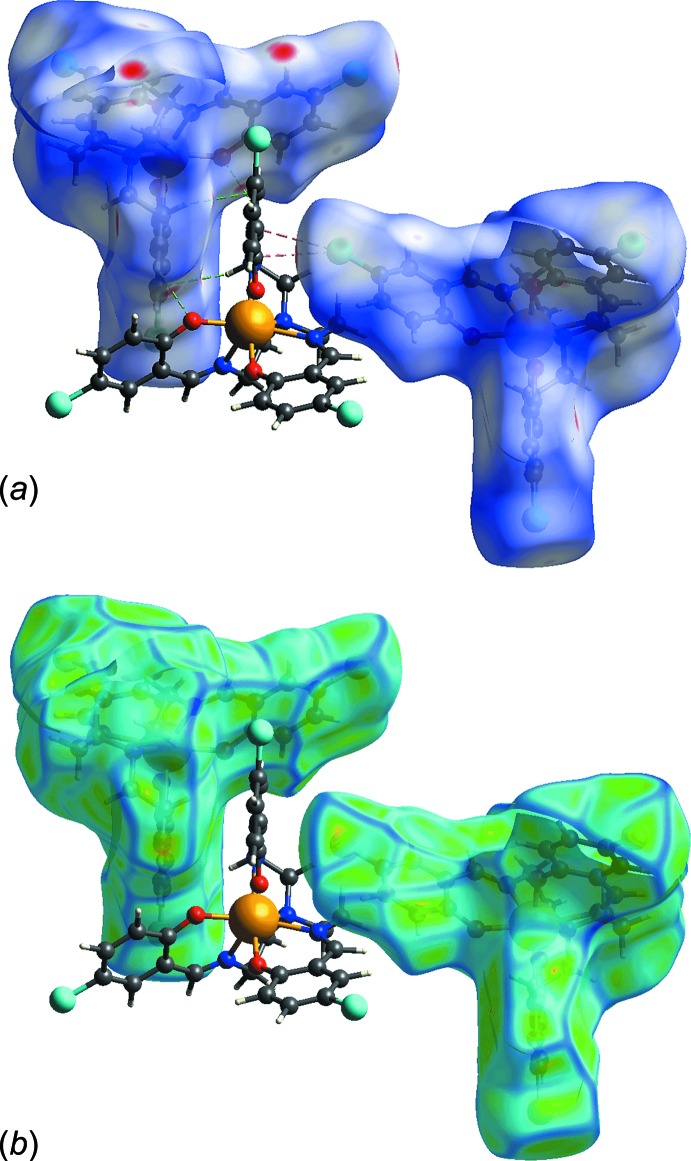
Connection of adjacent mol­ecules by C⋯H/H⋯C and O⋯H/H⋯O contacts in a shape complementary array mapped over the (*a*) Hirshfeld surface and (*b*) curvedness.

The Hirshfeld surface analysis was performed on (I) in order to gain better understanding, on a qu­anti­tative basis, of the different close inter­molecular contacts. The percentage contributions to the overall Hirshfeld surface are summarized in Table 3 ▸. Fig. 3 ▸ *a* shows a butterfish-like two-dimensional fingerprint plot for (I). In general, the diffuse region at the top-right corner of the plot may indicate relatively low packing efficiency which could be due to the absence of hydrogen bonding. A detailed analysis of the decomposed fingerprint plots reveals that close contacts resulting from C⋯H/H⋯C as well as O⋯H/H⋯O contacts are evident. These constitute *ca* 26 and 5%, respectively, of the overall inter­actions in the crystal, with *d*_e_ + *d*_i_ distances of ∼2.54 Å and ∼2.43 Å, respectively, Fig. 3 ▸ *b* and 3*c*. As seen from the images of Fig. 4 ▸, these inter­actions connect adjacent mol­ecules in such a way that the mol­ecules are arranged in a shape complementary array with an overall packing index of 69.6%. The end-on C—Cl⋯π(ar­yl) contacts appear as a focused area in the middle of the fingerprint plot delineated into Cl⋯C/C⋯Cl contacts, Fig. 3 ▸ *d*. Finally, the Cl⋯H/H⋯Cl inter­actions appear as the third most dominant inter­action, right after H⋯H and C⋯H/H⋯C, with an overall contribution of *ca* 24% to the Hirshfeld surface, despite the fact that these inter­actions are considered weak with contact distances greater than the sum of van der Waals radii. However, as seen from Fig. 3 ▸ *e*, these inter­actions are responsible for the appearance of the tails of the ‘butterfish-shape’.

## Database survey   

The structure of the unsubstituted Yb^III^ complex has been the subject of two independent determinations (Bernhardt *et al.*, 2001 ▸; Kanesato *et al.*, 2004 ▸). The mol­ecule exhibits a very similar coordination geometry except that it adopts crystallographic threefold symmetry. The Yb—O, Yb—N(imine) and Yb—N(amine) bond lengths, taken from Kanesato *et al.* (2004 ▸), are 2.168 (5), 2.433 (6) and 2.700 (7) Å, respectively, and follow the same trends as in (I), Table 1 ▸, and indeed are equal within experimental error.

A wider range of lanthanide (*Ln*) structures are available for comparison where the ligand is identical to that in (I), and each conforms to the conformation found in (I), approximating threefold symmetry. These fall into two crystal symmetries, *i.e. P*2_1_/*n*, for *Ln* = Sm (Kanesato *et al.*, 2001*a* ▸), Tb (Hu *et al.*, 2015 ▸) and Er (Pedersen *et al.*, 2014 ▸) [approximate reduced-cell parameters: *a* = 12.6 Å, *b* = 15.1 Å, *c* = 15.3 Å and γ = 110°] and *P*2_1_/*c*, for *Ln* = Gd (Kanesato *et al.*, 2001*b* ▸) and Yb in (I) [approximate reduced-cell parameters: *a* = 10.1 Å, *b* = 13.2 Å, *c* = 21.3 Å and β = 101°], having no obvious trends across the series. Salient bond-length data are collated in Table 4 ▸ for the five structures. As expected from the influence of the lanthanide contraction across the series *Ln* = Sm, Gd, Tb, Er and Yb, there is a systematic reduction in the *Ln*—O and *Ln*—N(imine) bond lengths. The only anomalous parameter might be the length of the Gd—N(amine) bond, *i.e*. 2.737 (8) Å, the relatively high standard uncertainty value notwithstanding.

## Synthesis and crystallization   

The Schiff base ligand, tris­{[(5-chloro­salicyl­idene)amino]­eth­yl}amine (Kanesato *et al.*, 2000 ▸; 0.56 g, 1 mmol) and tri­ethyl­amine (0.14 ml, 1.0 mmol) were taken in absolute ethanol (25 ml) and refluxed for 1 h. An ethano­lic solution (15 ml) of ytterbium(III) chloride hexa­hydrate (Sigma–Aldrich; 0.39 g, 1 mmol) was added to the mixture which was refluxed for 2 h and filtered. The filtrate was evaporated until a precipitate was obtained. The precipitate was recrystallized from ethanol solution and the by-product, tri­ethyl­ammonium chloride, was removed through filtration. Yellow needles of the title complex suitable for X-ray crystallographic studies were obtained from the slow evaporation of the filtrate. Yield: 0.55 g (75%). M.p.: 380– 382 K. IR (cm^−1^): 1628 (*s*) ν(C=N), 1517 (*m*), 1449 (*m*), 1392 (*m*) ν(–O—C=C–), 1158 (*m*) ν(C—O—C). Analysis calculated for C_27_H_24_Cl_3_N_4_O_3_Yb: C, 44.31; H, 3.31; N, 7.66%. Found: C, 44.63; H, 3.12; N, 7.92%.

## Refinement   

Crystal data, data collection and structure refinement details are summarized in Table 5 ▸. The carbon-bound H-atoms were placed in calculated positions (C—H = 0.95–0.99 Å) and were included in the refinement in the riding-model approximation, with *U*_iso_(H) set to 1.2*U*_eq_(C). Owing to poor agreement, one reflection, *i.e*. (7 0 4), was omitted from the final cycles of refinement. The maximum and minimum residual electron density peaks of 1.65 and 0.45 e Å^−3^, respectively, were located 0.84 and 1.51 Å from the Yb and N2 atoms, respectively.

## Supplementary Material

Crystal structure: contains datablock(s) I, global. DOI: 10.1107/S2056989016013748/hb7612sup1.cif

Structure factors: contains datablock(s) I. DOI: 10.1107/S2056989016013748/hb7612Isup2.hkl

CCDC reference: 1501230

Additional supporting information: crystallographic information; 3D view; checkCIF report

## Figures and Tables

**Table 1:** Selected geometric parameters (Å, °)

Yb—O1	2.1476 (16)	Yb—N2	2.4331 (19)
Yb—O2	2.1715 (16)	Yb—N3	2.439 (2)
Yb—O3	2.1608 (17)	Yb—N4	2.442 (2)
Yb—N1	2.677 (2)		
			
O1—Yb—N2	75.38 (6)	O1—Yb—N4	160.51 (7)
O2—Yb—N3	74.91 (6)	O2—Yb—N2	164.04 (7)
O3—Yb—N4	74.67 (6)	O3—Yb—N3	165.13 (7)

**Table 2:** Hydrogen-bond geometry (Å, °) *Cg*1 is the centroid of the C4–C9 ring.

*D*—H⋯*A*	*D*—H	H⋯*A*	*D*⋯*A*	*D*—H⋯*A*
C3—H3⋯O3^i^	0.95	2.55	3.369 (3)	144
C23—H23⋯O2^ii^	0.95	2.60	3.413 (3)	144
C2—H2*B*⋯*Cg*1^i^	0.99	2.88	3.744 (3)	146
C24—Cl3⋯*Cg*1^iii^	1.74 (1)	3.48 (1)	5.109 (3)	155 (1)

Symmetry codes: (i) 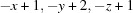; (ii) 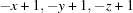; (iii) 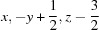.

**Table 3:** Percentage contribution of the different inter­molecular contacts to the Hirshfeld surface of (I)

Contact	% Contribution
H⋯H	35.3
C⋯H/H⋯C	25.9
Cl⋯H/H⋯Cl	23.9
Cl⋯C/C⋯Cl	5.9
O⋯H/H⋯O	5.0
Other	4.0

**Table 4:** Geometric data (Å, °) for (I) and literature analogues

Ln	Ln—O	Ln—N(imine)	Ln—N(amine)	Reference
Sm	2.237 (5)–2.243 (6)	2.545 (6)–2.562 (7)	2.778 (6)	Kanesato *et al.* (2001*a* ▸)
Gd	2.216 (7)–2.235 (7)	2.529 (8)–2.542 (8)	2.737 (8)	Kanesato *et al.* (2001*b* ▸)
Tb	2.206 (3)–2.218 (3)	2.495 (3)–2.510 (4)	2.748 (4)	Hu *et al.* (2015 ▸)
Er	2.175 (2)–2.1878 (19)	2.444 (2)–2.465 (2)	2.696 (2)	Pedersen *et al.* (2014 ▸)
Yb	2.1476 (16)–2.1715 (16)	2.4331 (19)–2.442 (2)	2.677 (2)	This work

**Table 5:** Experimental details

Crystal data	
Chemical formula	[Yb(C_27_H_24_Cl_3_N_4_O_3_)]
*M*_r_	731.89
Crystal system, space group	Monoclinic, *P*2_1_/*c*
Temperature (K)	100
*a*, *b*, *c* (Å)	10.0452 (2), 13.1510 (2), 21.0926 (4)
β (°)	101.221 (1)
*V* (Å^3^)	2733.16 (9)
*Z*	4
Radiation type	Mo *K*α
μ (mm^−1^)	3.75
Crystal size (mm)	0.37 × 0.06 × 0.04

Data collection	
Diffractometer	Bruker *SMART* *APEX* CCD
Absorption correction	Multi-scan (*SADABS*; Sheldrick, 1996 ▸)
*T*_min_, *T*_max_	0.337, 0.864
No. of measured, independent and observed [*I* > 2σ(*I*)] reflections	29037, 7882, 6584
*R*_int_	0.028
(sin θ/λ)_max_ (Å^−1^)	0.714

Refinement	
*R*[*F* ^2^ > 2σ(*F* ^2^)], *wR*(*F* ^2^), *S*	0.022, 0.055, 1.05
No. of reflections	7882
No. of parameters	343
H-atom treatment	H-atom parameters constrained
Δρ_max_, Δρ_min_ (e Å^−3^)	1.65, −0.45

Computer programs: *SMART* and *SAINT* (Bruker, 2008 ▸), *SHELXS97* (Sheldrick, 2008 ▸), *SHELXL2014* (Sheldrick, 2015 ▸), *ORTEP-3 for Windows* (Farrugia, 2012 ▸), *QMol* (Gans & Shalloway, 2001 ▸), *DIAMOND* (Brandenburg, 2006 ▸) and *publCIF* (Westrip, 2010 ▸).

## supplementary crystallographic information

### Crystal data

**Table:**

C_27_H_24_Cl_3_N_4_O_3_Yb	*F*(000) = 1436
*M**_r_* = 731.89	*D*_x_ = 1.779 Mg m^−^^3^
Monoclinic, *P*2_1_/*c*	Mo *K*α radiation, λ = 0.71073 Å
*a* = 10.0452 (2) Å	Cell parameters from 9874 reflections
*b* = 13.1510 (2) Å	θ = 2.5–30.5°
*c* = 21.0926 (4) Å	µ = 3.75 mm^−^^1^
β = 101.221 (1)°	*T* = 100 K
*V* = 2733.16 (9) Å^3^	Needle, yellow
*Z* = 4	0.37 × 0.06 × 0.04 mm

### Data collection

**Table:**

Bruker SMART APEX CCD diffractometer	7882 independent reflections
Radiation source: fine-focus sealed tube	6584 reflections with *I* > 2σ(*I*)
Graphite monochromator	*R*_int_ = 0.028
φ and ω scans	θ_max_ = 30.5°, θ_min_ = 1.8°
Absorption correction: multi-scan (SADABS; Sheldrick, 1996)	*h* = −14→13
*T*_min_ = 0.337, *T*_max_ = 0.864	*k* = −18→18
29037 measured reflections	*l* = −30→30

### Refinement

**Table:**

Refinement on *F*^2^	0 restraints
Least-squares matrix: full	Hydrogen site location: inferred from neighbouring sites
*R*[*F*^2^ > 2σ(*F*^2^)] = 0.022	H-atom parameters constrained
*wR*(*F*^2^) = 0.055	*w* = 1/[σ^2^(*F*_o_^2^) + (0.0236*P*)^2^ + 2.0547*P*] where *P* = (*F*_o_^2^ + 2*F*_c_^2^)/3
*S* = 1.05	(Δ/σ)_max_ = 0.004
7882 reflections	Δρ_max_ = 1.65 e Å^−^^3^
343 parameters	Δρ_min_ = −0.45 e Å^−^^3^

### Special details

**Table:**

Geometry. All esds (except the esd in the dihedral angle between two l.s. planes) are estimated using the full covariance matrix. The cell esds are taken into account individually in the estimation of esds in distances, angles and torsion angles; correlations between esds in cell parameters are only used when they are defined by crystal symmetry. An approximate (isotropic) treatment of cell esds is used for estimating esds involving l.s. planes.

### Fractional atomic coordinates and isotropic or equivalent isotropic displacement parameters (Å^2^)

**Table:**

	*x*	*y*	*z*	*U*_iso_*/*U*_eq_	
Yb	0.46352 (2)	0.75973 (2)	0.57731 (2)	0.01267 (3)	
Cl1	0.12533 (9)	1.28195 (5)	0.58909 (5)	0.0416 (2)	
Cl2	−0.02898 (6)	0.51120 (5)	0.75694 (3)	0.02505 (14)	
Cl3	0.36458 (6)	0.53255 (4)	0.23696 (3)	0.01926 (12)	
O1	0.29667 (17)	0.85663 (12)	0.58719 (8)	0.0167 (3)	
O2	0.33581 (17)	0.62842 (12)	0.58381 (8)	0.0164 (3)	
O3	0.38891 (18)	0.76939 (12)	0.47425 (8)	0.0159 (3)	
N1	0.7240 (2)	0.76956 (14)	0.63698 (10)	0.0151 (4)	
N2	0.5557 (2)	0.93115 (14)	0.58045 (10)	0.0154 (4)	
N3	0.4951 (2)	0.72213 (14)	0.69249 (10)	0.0148 (4)	
N4	0.6072 (2)	0.64484 (14)	0.52919 (10)	0.0148 (4)	
C1	0.7729 (2)	0.87631 (18)	0.64050 (12)	0.0175 (5)	
H1A	0.8721	0.8773	0.6424	0.021*	
H1B	0.7546	0.9087	0.6803	0.021*	
C2	0.7024 (2)	0.93543 (18)	0.58208 (13)	0.0184 (5)	
H2A	0.7338	1.0069	0.5851	0.022*	
H2B	0.7234	0.9053	0.5422	0.022*	
C3	0.4962 (3)	1.01700 (17)	0.58504 (12)	0.0171 (5)	
H3	0.5514	1.0762	0.5881	0.021*	
C4	0.3547 (3)	1.03227 (17)	0.58600 (11)	0.0164 (5)	
C5	0.3105 (3)	1.13402 (18)	0.58747 (12)	0.0208 (5)	
H5	0.3743	1.1880	0.5900	0.025*	
C6	0.1758 (3)	1.15543 (19)	0.58521 (14)	0.0246 (6)	
C7	0.0807 (3)	1.0776 (2)	0.58029 (14)	0.0252 (6)	
H7	−0.0127	1.0932	0.5772	0.030*	
C8	0.1222 (3)	0.97781 (19)	0.57992 (13)	0.0217 (5)	
H8	0.0562	0.9253	0.5766	0.026*	
C9	0.2601 (2)	0.95123 (17)	0.58435 (11)	0.0148 (4)	
C10	0.7392 (3)	0.72872 (18)	0.70367 (12)	0.0178 (5)	
H10A	0.8250	0.7538	0.7304	0.021*	
H10B	0.7432	0.6535	0.7026	0.021*	
C11	0.6200 (3)	0.76202 (18)	0.73361 (12)	0.0176 (5)	
H11A	0.6300	0.7347	0.7780	0.021*	
H11B	0.6163	0.8371	0.7357	0.021*	
C12	0.4136 (2)	0.67362 (17)	0.72151 (12)	0.0162 (5)	
H12	0.4367	0.6702	0.7673	0.019*	
C13	0.2894 (2)	0.62359 (17)	0.69055 (12)	0.0158 (5)	
C14	0.2018 (3)	0.59174 (17)	0.73117 (13)	0.0177 (5)	
H14	0.2243	0.6056	0.7762	0.021*	
C15	0.0842 (3)	0.54082 (18)	0.70628 (13)	0.0190 (5)	
C16	0.0522 (2)	0.51588 (17)	0.64104 (13)	0.0187 (5)	
H16	−0.0277	0.4782	0.6245	0.022*	
C17	0.1368 (2)	0.54595 (17)	0.60038 (13)	0.0176 (5)	
H17	0.1141	0.5285	0.5559	0.021*	
C18	0.2572 (2)	0.60244 (16)	0.62333 (12)	0.0157 (5)	
C19	0.8079 (3)	0.70835 (18)	0.60012 (12)	0.0178 (5)	
H19A	0.8942	0.6891	0.6289	0.021*	
H19B	0.8297	0.7496	0.5642	0.021*	
C20	0.7321 (3)	0.61317 (18)	0.57314 (12)	0.0176 (5)	
H20A	0.7890	0.5719	0.5495	0.021*	
H20B	0.7098	0.5714	0.6087	0.021*	
C21	0.5862 (2)	0.60742 (17)	0.47208 (12)	0.0164 (5)	
H21	0.6498	0.5586	0.4632	0.020*	
C22	0.4753 (2)	0.63213 (17)	0.41975 (11)	0.0137 (4)	
C23	0.4692 (3)	0.57793 (17)	0.36155 (12)	0.0164 (5)	
H23	0.5354	0.5275	0.3586	0.020*	
C24	0.3680 (3)	0.59760 (17)	0.30922 (12)	0.0161 (5)	
C25	0.2680 (3)	0.66999 (18)	0.31321 (12)	0.0175 (5)	
H25	0.1961	0.6813	0.2774	0.021*	
C26	0.2742 (2)	0.72490 (17)	0.36935 (12)	0.0159 (5)	
H26	0.2058	0.7739	0.3716	0.019*	
C27	0.3794 (2)	0.71038 (17)	0.42369 (12)	0.0147 (4)	

### Atomic displacement parameters (Å^2^)

**Table:**

	*U*^11^	*U*^22^	*U*^33^	*U*^12^	*U*^13^	*U*^23^
Yb	0.01552 (5)	0.00985 (5)	0.01280 (5)	0.00054 (4)	0.00315 (3)	−0.00017 (4)
Cl1	0.0456 (5)	0.0175 (3)	0.0678 (6)	0.0137 (3)	0.0262 (4)	0.0038 (3)
Cl2	0.0180 (3)	0.0273 (3)	0.0318 (4)	0.0009 (2)	0.0095 (3)	0.0094 (3)
Cl3	0.0252 (3)	0.0186 (3)	0.0143 (3)	−0.0015 (2)	0.0047 (2)	−0.0031 (2)
O1	0.0187 (9)	0.0123 (7)	0.0203 (9)	0.0016 (6)	0.0065 (7)	−0.0012 (6)
O2	0.0189 (9)	0.0128 (7)	0.0184 (9)	−0.0011 (6)	0.0058 (7)	−0.0016 (6)
O3	0.0211 (9)	0.0139 (7)	0.0124 (8)	0.0029 (6)	0.0027 (7)	0.0000 (6)
N1	0.0164 (10)	0.0133 (9)	0.0157 (10)	−0.0005 (7)	0.0031 (8)	0.0004 (7)
N2	0.0172 (10)	0.0142 (9)	0.0151 (10)	0.0007 (7)	0.0037 (8)	0.0014 (7)
N3	0.0163 (10)	0.0128 (9)	0.0146 (10)	−0.0001 (7)	0.0016 (8)	−0.0007 (7)
N4	0.0137 (9)	0.0132 (9)	0.0171 (10)	0.0015 (7)	0.0018 (8)	0.0007 (7)
C1	0.0164 (12)	0.0174 (11)	0.0182 (12)	−0.0035 (9)	0.0022 (10)	−0.0022 (9)
C2	0.0178 (12)	0.0159 (11)	0.0231 (13)	−0.0014 (9)	0.0078 (10)	0.0006 (9)
C3	0.0248 (13)	0.0125 (10)	0.0146 (12)	−0.0014 (9)	0.0052 (10)	0.0024 (8)
C4	0.0231 (12)	0.0147 (10)	0.0115 (11)	0.0031 (9)	0.0033 (9)	0.0024 (8)
C5	0.0295 (14)	0.0144 (11)	0.0202 (13)	0.0020 (10)	0.0093 (11)	0.0018 (9)
C6	0.0335 (15)	0.0140 (11)	0.0283 (15)	0.0103 (10)	0.0112 (12)	0.0022 (10)
C7	0.0219 (14)	0.0266 (13)	0.0283 (15)	0.0096 (10)	0.0077 (11)	0.0020 (11)
C8	0.0218 (13)	0.0210 (12)	0.0235 (14)	0.0016 (10)	0.0078 (11)	−0.0022 (10)
C9	0.0206 (12)	0.0146 (10)	0.0099 (11)	0.0028 (9)	0.0044 (9)	0.0001 (8)
C10	0.0170 (11)	0.0192 (11)	0.0162 (12)	0.0001 (9)	0.0008 (9)	0.0014 (9)
C11	0.0177 (11)	0.0182 (11)	0.0157 (11)	−0.0056 (9)	0.0001 (9)	0.0004 (9)
C12	0.0196 (12)	0.0135 (10)	0.0160 (12)	0.0026 (9)	0.0042 (9)	0.0000 (8)
C13	0.0169 (12)	0.0115 (10)	0.0190 (12)	0.0012 (8)	0.0038 (9)	0.0022 (8)
C14	0.0195 (12)	0.0146 (10)	0.0202 (13)	0.0024 (9)	0.0066 (10)	0.0023 (9)
C15	0.0176 (12)	0.0145 (10)	0.0270 (14)	0.0038 (9)	0.0096 (10)	0.0071 (9)
C16	0.0133 (11)	0.0133 (10)	0.0285 (14)	0.0009 (8)	0.0021 (10)	0.0015 (9)
C17	0.0172 (12)	0.0132 (10)	0.0222 (13)	0.0035 (9)	0.0030 (10)	−0.0017 (9)
C18	0.0153 (11)	0.0085 (9)	0.0236 (13)	0.0025 (8)	0.0043 (10)	0.0001 (8)
C19	0.0151 (11)	0.0197 (11)	0.0189 (12)	0.0020 (9)	0.0039 (9)	−0.0003 (9)
C20	0.0181 (12)	0.0167 (11)	0.0165 (12)	0.0047 (9)	0.0000 (9)	−0.0007 (9)
C21	0.0163 (12)	0.0136 (10)	0.0195 (12)	0.0022 (8)	0.0037 (10)	0.0006 (8)
C22	0.0138 (11)	0.0135 (10)	0.0142 (11)	−0.0007 (8)	0.0034 (9)	0.0002 (8)
C23	0.0191 (12)	0.0133 (10)	0.0173 (12)	0.0018 (9)	0.0049 (10)	−0.0012 (8)
C24	0.0231 (12)	0.0131 (10)	0.0128 (11)	−0.0029 (9)	0.0052 (9)	−0.0018 (8)
C25	0.0197 (12)	0.0168 (11)	0.0152 (12)	0.0003 (9)	0.0014 (10)	0.0019 (9)
C26	0.0167 (11)	0.0141 (10)	0.0160 (11)	0.0026 (8)	0.0008 (9)	0.0011 (8)
C27	0.0171 (11)	0.0120 (10)	0.0163 (11)	−0.0015 (8)	0.0059 (9)	0.0010 (8)

### Geometric parameters (Å, º)

**Table:**

Yb—O1	2.1476 (16)	C7—H7	0.9500
Yb—O2	2.1715 (16)	C8—C9	1.414 (3)
Yb—O3	2.1608 (17)	C8—H8	0.9500
Yb—N1	2.677 (2)	C10—C11	1.522 (4)
Yb—N2	2.4331 (19)	C10—H10A	0.9900
Yb—N3	2.439 (2)	C10—H10B	0.9900
Yb—N4	2.442 (2)	C11—H11A	0.9900
Cl1—C6	1.746 (2)	C11—H11B	0.9900
Cl2—C15	1.749 (3)	C12—C13	1.449 (3)
Cl3—C24	1.742 (2)	C12—H12	0.9500
O1—C9	1.295 (3)	C13—C14	1.406 (3)
O3—C27	1.307 (3)	C13—C18	1.419 (3)
O2—C18	1.300 (3)	C14—C15	1.371 (4)
N1—C1	1.484 (3)	C14—H14	0.9500
N1—C10	1.485 (3)	C15—C16	1.390 (4)
N1—C19	1.489 (3)	C16—C17	1.378 (4)
N2—C3	1.290 (3)	C16—H16	0.9500
N2—C2	1.469 (3)	C17—C18	1.421 (3)
N3—C12	1.283 (3)	C17—H17	0.9500
N3—C11	1.476 (3)	C19—C20	1.517 (3)
N4—C21	1.280 (3)	C19—H19A	0.9900
N4—C20	1.468 (3)	C19—H19B	0.9900
C1—C2	1.511 (3)	C20—H20A	0.9900
C1—H1A	0.9900	C20—H20B	0.9900
C1—H1B	0.9900	C21—C22	1.445 (3)
C2—H2A	0.9900	C21—H21	0.9500
C2—H2B	0.9900	C22—C23	1.410 (3)
C3—C4	1.440 (3)	C22—C27	1.423 (3)
C3—H3	0.9500	C23—C24	1.372 (3)
C4—C5	1.412 (3)	C23—H23	0.9500
C4—C9	1.424 (3)	C24—C25	1.398 (3)
C5—C6	1.374 (4)	C25—C26	1.378 (3)
C5—H5	0.9500	C25—H25	0.9500
C6—C7	1.390 (4)	C26—C27	1.412 (3)
C7—C8	1.377 (4)	C26—H26	0.9500
			
O1—Yb—N2	75.38 (6)	O1—C9—C8	120.4 (2)
O2—Yb—N3	74.91 (6)	O1—C9—C4	122.4 (2)
O3—Yb—N4	74.67 (6)	C8—C9—C4	117.2 (2)
O1—Yb—N4	160.51 (7)	N1—C10—C11	110.14 (19)
O2—Yb—N2	164.04 (7)	N1—C10—H10A	109.6
O3—Yb—N3	165.13 (7)	C11—C10—H10A	109.6
O1—Yb—O3	86.46 (6)	N1—C10—H10B	109.6
O1—Yb—O2	89.08 (6)	C11—C10—H10B	109.6
O3—Yb—O2	90.96 (6)	H10A—C10—H10B	108.1
O3—Yb—N2	91.64 (6)	N3—C11—C10	107.5 (2)
O1—Yb—N3	88.64 (7)	N3—C11—H11A	110.2
N2—Yb—N3	100.70 (7)	C10—C11—H11A	110.2
O2—Yb—N4	86.47 (6)	N3—C11—H11B	110.2
N2—Yb—N4	109.39 (7)	C10—C11—H11B	110.2
N3—Yb—N4	108.41 (7)	H11A—C11—H11B	108.5
O1—Yb—N1	129.46 (6)	N3—C12—C13	125.8 (2)
O3—Yb—N1	125.89 (6)	N3—C12—H12	117.1
O2—Yb—N1	122.97 (6)	C13—C12—H12	117.1
N2—Yb—N1	67.08 (6)	C14—C13—C18	120.2 (2)
N3—Yb—N1	67.39 (7)	C14—C13—C12	116.5 (2)
N4—Yb—N1	67.84 (6)	C18—C13—C12	123.2 (2)
C9—O1—Yb	141.74 (16)	C15—C14—C13	120.5 (2)
C27—O3—Yb	137.88 (15)	C15—C14—H14	119.8
C18—O2—Yb	133.58 (15)	C13—C14—H14	119.8
C1—N1—C10	108.82 (19)	C14—C15—C16	120.7 (2)
C1—N1—C19	108.78 (19)	C14—C15—Cl2	119.3 (2)
C10—N1—C19	109.54 (18)	C16—C15—Cl2	120.01 (19)
C1—N1—Yb	110.66 (14)	C17—C16—C15	119.8 (2)
C10—N1—Yb	109.81 (14)	C17—C16—H16	120.1
C19—N1—Yb	109.20 (14)	C15—C16—H16	120.1
C3—N2—C2	116.2 (2)	C16—C17—C18	121.7 (2)
C3—N2—Yb	129.32 (17)	C16—C17—H17	119.2
C2—N2—Yb	114.26 (14)	C18—C17—H17	119.2
C12—N3—C11	116.4 (2)	O2—C18—C13	122.7 (2)
C12—N3—Yb	127.19 (17)	O2—C18—C17	120.1 (2)
C11—N3—Yb	116.37 (15)	C13—C18—C17	117.1 (2)
C21—N4—C20	116.7 (2)	N1—C19—C20	110.4 (2)
C21—N4—Yb	128.79 (16)	N1—C19—H19A	109.6
C20—N4—Yb	114.50 (15)	C20—C19—H19A	109.6
N1—C1—C2	110.35 (19)	N1—C19—H19B	109.6
N1—C1—H1A	109.6	C20—C19—H19B	109.6
C2—C1—H1A	109.6	H19A—C19—H19B	108.1
N1—C1—H1B	109.6	N4—C20—C19	107.91 (19)
C2—C1—H1B	109.6	N4—C20—H20A	110.1
H1A—C1—H1B	108.1	C19—C20—H20A	110.1
N2—C2—C1	107.95 (19)	N4—C20—H20B	110.1
N2—C2—H2A	110.1	C19—C20—H20B	110.1
C1—C2—H2A	110.1	H20A—C20—H20B	108.4
N2—C2—H2B	110.1	N4—C21—C22	126.4 (2)
C1—C2—H2B	110.1	N4—C21—H21	116.8
H2A—C2—H2B	108.4	C22—C21—H21	116.8
N2—C3—C4	126.6 (2)	C23—C22—C27	120.1 (2)
N2—C3—H3	116.7	C23—C22—C21	116.6 (2)
C4—C3—H3	116.7	C27—C22—C21	123.2 (2)
C5—C4—C9	119.9 (2)	C24—C23—C22	120.3 (2)
C5—C4—C3	116.6 (2)	C24—C23—H23	119.8
C9—C4—C3	123.5 (2)	C22—C23—H23	119.8
C6—C5—C4	120.3 (2)	C23—C24—C25	120.6 (2)
C6—C5—H5	119.8	C23—C24—Cl3	119.79 (19)
C4—C5—H5	119.8	C25—C24—Cl3	119.66 (19)
C5—C6—C7	120.6 (2)	C26—C25—C24	119.7 (2)
C5—C6—Cl1	119.1 (2)	C26—C25—H25	120.1
C7—C6—Cl1	120.2 (2)	C24—C25—H25	120.1
C8—C7—C6	119.8 (3)	C25—C26—C27	121.9 (2)
C8—C7—H7	120.1	C25—C26—H26	119.1
C6—C7—H7	120.1	C27—C26—H26	119.1
C7—C8—C9	121.9 (2)	O3—C27—C26	120.4 (2)
C7—C8—H8	119.0	O3—C27—C22	122.3 (2)
C9—C8—H8	119.0	C26—C27—C22	117.3 (2)
			
C10—N1—C1—C2	−153.1 (2)	C13—C14—C15—Cl2	−174.95 (18)
C19—N1—C1—C2	87.6 (2)	C14—C15—C16—C17	−2.5 (4)
Yb—N1—C1—C2	−32.3 (2)	Cl2—C15—C16—C17	175.23 (18)
C3—N2—C2—C1	118.2 (2)	C15—C16—C17—C18	0.0 (3)
Yb—N2—C2—C1	−57.5 (2)	Yb—O2—C18—C13	−36.9 (3)
N1—C1—C2—N2	58.5 (3)	Yb—O2—C18—C17	146.44 (17)
C2—N2—C3—C4	−178.6 (2)	C14—C13—C18—O2	−178.7 (2)
Yb—N2—C3—C4	−3.6 (4)	C12—C13—C18—O2	−1.5 (3)
N2—C3—C4—C5	−176.3 (2)	C14—C13—C18—C17	−1.9 (3)
N2—C3—C4—C9	2.5 (4)	C12—C13—C18—C17	175.2 (2)
C9—C4—C5—C6	−2.1 (4)	C16—C17—C18—O2	179.0 (2)
C3—C4—C5—C6	176.8 (2)	C16—C17—C18—C13	2.2 (3)
C4—C5—C6—C7	−1.2 (4)	C1—N1—C19—C20	−158.2 (2)
C4—C5—C6—Cl1	178.5 (2)	C10—N1—C19—C20	83.0 (2)
C5—C6—C7—C8	2.3 (4)	Yb—N1—C19—C20	−37.3 (2)
Cl1—C6—C7—C8	−177.3 (2)	C21—N4—C20—C19	126.9 (2)
C6—C7—C8—C9	−0.1 (4)	Yb—N4—C20—C19	−54.7 (2)
Yb—O1—C9—C8	165.02 (19)	N1—C19—C20—N4	61.0 (3)
Yb—O1—C9—C4	−15.5 (4)	C20—N4—C21—C22	−176.0 (2)
C7—C8—C9—O1	176.5 (2)	Yb—N4—C21—C22	5.9 (4)
C7—C8—C9—C4	−3.1 (4)	N4—C21—C22—C23	−177.3 (2)
C5—C4—C9—O1	−175.4 (2)	N4—C21—C22—C27	6.0 (4)
C3—C4—C9—O1	5.8 (4)	C27—C22—C23—C24	−2.4 (3)
C5—C4—C9—C8	4.1 (3)	C21—C22—C23—C24	−179.2 (2)
C3—C4—C9—C8	−174.7 (2)	C22—C23—C24—C25	−1.4 (4)
C1—N1—C10—C11	81.9 (2)	C22—C23—C24—Cl3	178.20 (18)
C19—N1—C10—C11	−159.25 (19)	C23—C24—C25—C26	2.6 (4)
Yb—N1—C10—C11	−39.3 (2)	Cl3—C24—C25—C26	−176.95 (19)
C12—N3—C11—C10	129.8 (2)	C24—C25—C26—C27	0.0 (4)
Yb—N3—C11—C10	−51.8 (2)	Yb—O3—C27—C26	151.26 (19)
N1—C10—C11—N3	59.8 (2)	Yb—O3—C27—C22	−31.0 (4)
C11—N3—C12—C13	−176.5 (2)	C25—C26—C27—O3	174.3 (2)
Yb—N3—C12—C13	5.3 (3)	C25—C26—C27—C22	−3.6 (3)
N3—C12—C13—C14	−168.2 (2)	C23—C22—C27—O3	−173.0 (2)
N3—C12—C13—C18	14.6 (4)	C21—C22—C27—O3	3.5 (4)
C18—C13—C14—C15	−0.6 (3)	C23—C22—C27—C26	4.8 (3)
C12—C13—C14—C15	−177.9 (2)	C21—C22—C27—C26	−178.6 (2)
C13—C14—C15—C16	2.8 (4)		

### Hydrogen-bond geometry (Å, º)

Cg1 is the centroid of the C4–C9 ring.

**Table:**

*D*—H···*A*	*D*—H	H···*A*	*D*···*A*	*D*—H···*A*
C3—H3···O3^i^	0.95	2.55	3.369 (3)	144
C23—H23···O2^ii^	0.95	2.60	3.413 (3)	144
C2—H2*B*···*Cg*1^i^	0.99	2.88	3.744 (3)	146
C24—Cl3···*Cg*1^iii^	1.74 (1)	3.48 (1)	5.109 (3)	155 (1)

Symmetry codes: (i) −*x*+1, −*y*+2, −*z*+1; (ii) −*x*+1, −*y*+1, −*z*+1; (iii) *x*, −*y*+1/2, *z*−3/2.
